# Triptolide attenuates inhibition of ankylosing spondylitis-derived mesenchymal stem cells on the osteoclastogenesis through modulating exosomal transfer of circ-0110634

**DOI:** 10.1016/j.jot.2022.05.007

**Published:** 2022-09-16

**Authors:** Wei Ji, Yueyang Lu, Zhuoyi Ma, Ke Gan, Yan Liu, Yue Cheng, Junliang Xu, Shijia Liu, Yunke Guo, Shanhang Han, Zengyan Zhao, Hanmei Xu, Weiyan Qi

**Affiliations:** a Department of Rheumatology, Affiliated Hospital of Nanjing University of Chinese Medicine, Nanjing, 210023, Jiangsu, China; b School of Medicine and Holistic Integrative Medicine, Nanjing University of Chinese Medicine, Nanjing, 210033, Jiangsu, China; c The Engineering Research Center of Synthetic Polypeptide Drug Discovery and Evaluation, Jiangsu Province, China Pharmaceutical University, Nanjing, 210033, Jiangsu, China; d Department of Pharmacy, Affiliated Hospital of Nanjing University of Chinese Medicine, Nanjing, 210023, Jiangsu, China; e China Pharmaceutical University, Nanjing, 210033, Jiangsu, China

**Keywords:** Circ-0110634, Triptolide, Osteoclastogenesis, Ubiquitination, TRAF2

## Abstract

**Background:**

Ankylosing spondylitis (AS) is featured by chronic inflammation of the sacroiliac joints and spine as well as pathological new bone formation. Osteoclastogenesis is a critical part in the development of bone formation. Circular RNAs (circRNAs) are recent research hotspot in the RNA field while rarely reported in osteoclastogenesis.

**Methods:**

AS mesenchymal stem cells (ASMSCs) and healthy donor mesenchymal stem cells (HDMSCs) were co-cultured with peripheral blood mononuclear cells (PBMCs). RT-qPCR was applied to detect the expression level of circ-0110634 in different exosomes. TRAP staining and TRAP activity detection were performed to identify the effect of circ-0110634 overexpression on osteoclastogenesis. Bioinformatics analysis and mechanism investigation were conducted to explore the downstream molecular mechanism of circ-0110634.

**Results:**

The effect of ASMSCs on PBMCs osteoclastogenesis is weaker than that of HDMSCs. Circ-0110634 had higher expression in ASMSCs exosomes than HDMSCs exosomes. Circ-0110634 overexpression suppressed the osteoclastogenesis. Circ-0110634 bound to both TNF receptor associated factor 2 (TRAF2) and tumor necrosis factor receptor II (TNFRII). Circ-0110634 also accelerated the dimerization of TRAF2 to induce TRAF2 ubiquitination and degradation. Circ-0110634 repressed the interplay between TRAF2 and TNFRII to inactivate the nuclear factor-κB (NF-κB) and mitogen-activated protein kinases (MAPK) pathways. Triptolide promoted the osteoclastogenesis of ASMSCs exosomes-treated PBMCs via decreasing the exosomal transference of circ-0110634 in a dose-dependent manner. Consistently, triptolide treatment stimulated osteoclastogenesis to alleviate the arthritis of DBA/1 mice through suppressing circ-0110634.

**Conclusion:**

Our study confirmed that triptolide targets circ-0110634 to ease the burden of AS patients.

**The Translational potential of this article:**

This study suggests triptolide targets circ-0110634 to regulate osteoclastogenesis, which provides a novel potential target in triptolide treatment for AS patients.

## 1 Introduction

Ankylosing spondylitis (AS) is a typical inflammatory rheumatic disease occurred with pathological new bone formation, ankylosis and chronic inflammation in spine [1]. It can cause functional impairments and reduction of life quality [2]. The detailed disease mechanism of AS is still unclear as it is a complex multifactorial process involved with genetic and environmental factors as well as autoimmune responses [3]. Pathological osteogenesis is one of the main features of AS [4], but the underlying mechanism remains poorly recognized. More importantly, the early diagnosis of pathological osteogenesis is currently difficult, and effective treatment for pathological osteogenesis has not been defined yet [5]. Therefore, it is of significance to understand the pathogenesis of the osteogenesis that happens in AS and to identify effective strategies for early diagnosis and treatment. As we all know, mesenchymal stem cells (MSCs) are the primary source of osteoblasts [6]. As reported previously, MSCs from AS patients (ASMSCs) possesses a stronger capacity to differentiate into osteoblasts than MSCs from healthy donors (HDMSCs), suggesting the implication of ASMSCs in pathological osteogenesis [7]. However, the regulatory mechanism of ASMSCs on osteoclastogenesis is blurry.

Exosomes are mini bubbles around 40–100 ​nm long in diameter. They are mainly originated from multi-vesicular bodies formed by intracellular lysosomal microparticles and produced by various types of cells [[8], [9], [10]]. Exosomes have crucial functions in diseases by transferring proteins or RNAs to target cells. It has been reported that adipose mesenchymal stem cell-derived exosomes attenuate hypoxia/serum deprivation-induced osteocyte apoptosis and osteocyte-mediated osteoclastogenesis [11]. In this study, we also investigated whether ASMSCs carried exosomes to affect osteoclastogenesis.

Circular RNAs (circRNAs) are a class of non-coding RNAs with closed ring structure and are not affected by RNA exonuclease [12]. With the development of RNA sequencing techniques along with bioinformatics predictions, numerous circRNAs have been identified to have the features of developmental regulation, localization and tissue-specific expression [13]. Recent studies have demonstrated that circRNAs play crucial roles in the process of pathological osteogenesis [14]. Hsa_circ_0074834 contributes to the osteogenesis via acting as a ceRNA for miR-942–5p [15]. CircRNA_33287 increases the osteogenic differentiation via regulating the miR-214–3p/Runx3 axis [16]. Recently, circRNAs have been reported to exert regulatory function on their host genes [17]. TNF receptor superfamily member 1B (TNFRSF1B) has been reported as an osteogenesis-related differential expressed gene [18], and it is involved in miR-125a-5p promoted osteoclastogenesis [19]. Therefore, we intended to investigate the role of circRNA originated from TNFRSF1B gene in osteoclastogenesis.

Triptolide is a kind of traditional medicine, bitter and cold, with the effect of clearing heat, detoxifying dispel wind, clearing collaterals, relaxing muscles and promoting blood circulation. It is widely used in the treatment of rheumatoid arthritis, nephritis and lupus erythematosus [20,21]. Notably, triptolide can inhibit the osteoclastogenesis and serve as a potential treatment for osteoporosis [22]. However, whether triptolide affects the osteoclastogenesis to attenuate AS needs to be further elucidated in our study.

In our study, we aimed to investigate the function of circ-0110634 in osteoclastogenesis of peripheral blood mononuclear cells (PBMCs). Besides, we also wanted to verify the existence of exosomes and explore the role of exosomes in osteoclastogenesis of PBMCs. Moreover, the relation between circ-0110634 and triptolide in osteoclastogenesis was also probed.

## 2 Materials and methods

### 2.1 Reagents

DMSO, 2 ​μg/ml of Actinomycin D (Act D), 5 ​μg/ml of cycloheximide (CHX), 4 ​mM of aspirin, and 300 ​μM of sulfasalazine, were purchased from Sigma–Aldrich (Miamisburg, OH, USA). Twenty-five ng/ml of human RANKL and 25 ​ng/ml of recombinant human macrophage colony stimulating factor (M-CSF) were purchased from R&D Systems Inc (Minneapolis, Minnesota, USA) to induce osteoclastic differentiation. Three U/μg of RNase R was purchased from Epicentre Technologies (Madison, WI, USA). MG132 (10 ​μM) was purchased from Selleck Chemicals (Houston, TX, USA). Infliximab was purchased from Shanghai Teramabs Biotechnology Co., Ltd (Shanghai, China). Triptolide at 7 ​nM and 14 ​nM were purchased from EFEBIO Co., Ltd (Shanghai, China). Methotrexate was purchased from Minapharm Pharmaceuticals (Cairo, Egypt).

### 2.2 Patients and controls

One healthy donor and one AS patient were recruited in this study, and both of them signed the informed consents. This study was conducted under the approval from the Ethical Committee of our hospital.

### 2.3 Cell isolation and culture

The AS patient discontinued the treatment 14 days before the bone marrow puncture by skilled allied health professionals, for minimizing the effect of therapy. MSCs were instantly isolated from bone marrow samples of AS patient or healthy control via density gradient centrifugation, and then maintained in DMEM (Gibco, Rockville, MD, USA) with 10% fetal bovine serum (FBS; Gibco) in 5% CO_2_ at 37 ​°C. The culture medium was replaced every three days, and MSCs were passaged at about 90% cell confluence. The MSCs at passage 3–4 were collected for experiments.

### 2.4 Flow cytometry

HDMSCs and ASMSCs were treated with 0.25% trypsin and 0.53 ​mM EDTA (Gibco) for digestion, and then centrifuged. After that, cells were re-suspended in phosphate buffered saline (PBS) and cultured for half an hour with the specific antibodies to CD14-phycoerythrin (PE), CD45-FITC, HLA DR-PE, CD29-PE, CD44-FITC or CD105-FITC. To identify the phenotypes of HDMSCs and ASMSCs, flow cytometry was undertaken following the established protocol (BD Biosciences, Franklin Lakes, NJ, USA). The assay was taken in triplicate.

### 2.5 Tartrate-resistant acid phosphatase (TRAP) staining

For TRAP staining assay, the Acid Phosphatase Kit was procured from Sigma–Aldrich. After three days of cell culture, the PBMCs samples in each group were collected and fixed in Fixative Solution for 30 ​s at room temperature, washed in deionized water, and then cultured with TRAP staining solution. The plates were incubated for 1 ​h in the dark at 37 ​°C, subsequently rinsed for three times in distilled water. TRAP positive multinuclear cells were finally observed using inverted microscope (Nikon, Tokyo, Japan). TRAP activity was detected in culture medium. The assay was taken in triplicate.

### 2.6 Determination of bone resorption pits

PBMCs samples in each group were cultured in proliferation medium and seeded on the bovine bone slices of 24-well plates (5 ​× ​10^5^ ​cells per well) for one-day. After switching to differentiation medium for 3 days, the bovine bone slices were subjected to ultra-sonication using 1 ​mol/L of NH_4_OH for removing the adherent cells, and 0.1% toluidine blue solution was added for staining. The bone resorption pits area versus the total bone area in each slice was detected by Image Pro Plus 6.2 software (Media Cybernetics, Rockville, Maryland, USA). The assay was taken in triplicate.

### 2.7 Real-time quantitative RT-PCR

The total RNA was isolated from the processed cells by the usage of TRIzol Reagent (Invitrogen, Carlsbad CA, USA). After that, complementary DNA (cDNA) synthesis was undertaken in presence of PrimeScript Reverse Transcriptase Kit (Takara, Shiga, Japan). SYBR Green PCR Kit (Takara) was employed for qPCR on ABI Prism 7900HT sequence detector (Applied Biosystems, Foster City, CA, USA). Gene expression was calculated with the comparative change-in-cycle method (ΔΔCt) and normalized to GAPDH. The assay was taken in triplicate.

### 2.8 Cell transfection

1 ​× ​10^6^ ​cells were collected from various treatment groups and seeded into each well of 6-well plates. The specific shRNAs to circ-0110634 and non-specific shRNAs were constructed by GenePharma (Shanghai, China). For overexpressing the expression of circ-0110634, TRAF2 and TNFRSF1B, the pcDNA3.1 (+) CircRNA Mini Vector and pcDNA3.1 vectors were procured from Genechem (Shanghai, China), as well the empty vectors as negative controls (NC). The transfection was conducted for 48 ​h by using Lipofectamine 3000 (Invitrogen). The assay was taken in triplicate.

### 2.9 Western blot

After lysing in RIPA buffer, the collected total protein was separated on 12% SDS-PAGE and shifted onto PVDF membranes. 5% nonfat milk powder was added for blocking membranes. Next, the membrane was probed all night at 4 ​°C with primary antibodies against loading control GAPDH (ab8245, 1/1000; Abcam, Cambridge, MA, USA), TRAP (ab65854, 1/1000; Abcam), NFATc1 (ab175134, 1/1000; Abcam), TRAF3 (ab36988, 1/1000; Abcam), TRAF1 (ab129279, 1/1000; Abcam), TRADD (ab110644, 1/1000; Abcam), TRAF2 (ab230795, 1/1000; Abcam), ADAM17 (ab39162, 1/1000; Abcam), TNFRII (ab8161, 1/1000; Abcam), TNFRSF1A (ab19139, 1/1000; Abcam), p-IKB (ab133462, 1/1000; Abcam), IKB (ab32518, 1/1000; Abcam), TNFRSF11 (NB100-56508, 1/1000; Novus Biologicals, Littleton, CO, USA), p-IKKα (ab38515, 1/1000; Abcam), IKKα (ab32041, 1/1000; Abcam), p-IKKβ (ab59195, 1/1000; Abcam), IKKβ (ab124957, 1/1000; Abcam), p-Erk (ab214036, 1/1000; Abcam), Erk (ab184699, 1/10000; Abcam), p-MEK (ab96379, 1/1000; Abcam), MEK (ab32091, 1/1000; Abcam). On the following day, the HRP-secondary antibodies were added for 2 ​h at room temperature. The protein density was examined by the ECL luminous liquid (Pierce, Rockford, IL, USA). The assay was taken in triplicate.

### 2.10 Exosome isolation

ASMSCs and HDMSCs were cultured in exosome-free medium for the isolation of exosomes from cell supernatants. 3 days later, exosomes were isolated and purified by use of Total Exosome Isolation Reagent (Thermo Fisher Scientific, Waltham, MA, USA), as instructed. Exosomes derived from ASMSCs and HDMSCs were named as HDMSCs exo and ASMSCs exo. The assay was taken in triplicate.

### 2.11 Transmission electron microscope (TEM) analysis

TEM analysis was employed to identify the morphology of HDMSCs exo and ASMSCs exo on Hitachi H-7650 TEM (Hitachi, Tokyo, Japan). Images were all captured using digital camera (Olympus, Tokyo, Japan). The assay was taken in triplicate.

### 2.12 Dynamic light scattering (DLS) analysis

DLS analysis was utilized to detect the size of nanoparticles. Samples of HDMSCs exo or ASMSCs exo were completely homogenized, and then 50 ​μl of sample was diluted with 1000 ​μl of distilled water. 100 ​ml of sample was detected in single-use polystyrene half-micro cuvettes. Next, the collected data were analyzed by Dispersion Technology Software. The assay was taken in triplicate.

### 2.13 Exosome uptake

For tracking exosome internalization, the exosomes were fluorescently labeled with the PKH26 red membrane dye (Sigma–Aldrich), following the standard method. The labeled exosomes were added into PBMCs and cultured for 6 ​h. After DAPI staining (Beyotime, Shanghai, China), Leica TCS SP5 II laser scanning confocal microscope (Leica Camera AG Inc., Frankfurt, Germany) was applied for observing the uptake of labeled exosomes by the recipient PBMCs. The assay was taken in triplicate.

### 2.14 Co-culture

To conduct the transwell co-culture, SaOS-2 ​cells (ATCC; Manassas, VA, USA) were acquired for generating the osteoblast-derived extracellular matrix. PBMCs were seeded on the SaOS-2 matrix in bottom compartment, and then HDMSCs or ASMSCs were seeded on the membrane of the 24-well transwell plates (3 ​μm; BD Biosciences, Franklin Lakes, NJ, USA). After the separate culture between PBMCs and HDMSCs or ASMSCs on SaOS-2 matrix, their supernatant was collected at 14-day of co-culture for analysis. Besides, PBMCs were co-cultured similarly with HDMSCs exo or ASMSCs exo, and collected for further analysis. The assay was taken in triplicate.

### 2.15 Fluorescent in situ hybridization (FISH)

The specific FISH probe targeting circ-0110634 was designed and constructed by Ribobio (Guangzhou, China). After indicated treatment, cells were reaped and cultured with FISH probe in the hybridization buffer. Hoechst solution (Beyotime) was used for counter-staining cell nucleus. The images were acquired using fluorescence microscope (Olympus). The assay was taken in triplicate.

### 2.16 Nuclear cytoplasm fractionation

The fractionation of cell nucleus and cell cytoplasm was studied by use of PARIS™ Kit (Invitrogen). HDMSCs and ASMSCs were centrifuged prior to lysing in cell fractionation buffer and cell disruption buffer. The content of circ-0110634, GAPDH and U6 were all detected in cell fractions using RT-qPCR. The assay was taken in triplicate.

### 2.17 Co-immunoprecipitation (Co-IP)

Cell lysates were acquired from the treated cells using IP lysis buffer, and then cultured with the specific antibodies and negative control IgG antibody all night at 4 ​°C. After mixing with Protein A/G PLUSAgarose (Santa Cruz Biotechnology, Dallas, TX, USA), the antigen–antibody mixture was acquired and incubated with magnetic beads. Samples were then washed in IP lysis buffer and eluted for western blot analysis. The assay was taken in triplicate.

### 2.18 RNA pull-down

RNA pull down assay was studied in presence of Pierce Magnetic RNA-Protein Pull-Down Kit (Thermo Fisher Scientific, Waltham, MA, USA). HEK-293T cells (ATCC, Manassas, VA, USA) were used. The cell protein extracts were prepared using RIPA lysis buffer, and then cultivated with the circ-0110634 probe or its antisense probe. 30 ​μl of magnetic beads was subsequently added. The final mixture was detected by mass spectrometry and western blot. The assay was taken in triplicate.

### 2.19 RNA immunoprecipitation (RIP)

RIP assay was studied with application of Magna RIP™ RNA-Binding Protein Immunoprecipitation Kit (Millipore, Bedford, MA, USA), using the antibodies against TNFRII, TRAF2 and control IgG. After lysing in RIP lysis buffer, the cell lysates were cultured with antibodies-bound magnetic beads in RIP buffer, followed by RT-qPCR analysis. The assay was taken in triplicate.

### 2.20 Animal studies

Aging male DBA/1 mice provided by Janvier Laboratory (Le Genest St Isle, France) were used as the spontaneous ankylosis model as previously described [23]. Mice from different litters were mixed and divided into three groups with the mean age of ten weeks each group. The first group of mice were subjected to intraperitoneal injection of PBS, the second group of mice were treated with triptolide, and the third group of mice were treated with equivalent dose of triptolide plus 30 ​μg of pcDNA3.1/circ-0110634. The dose of triptolide for mice was 20 ​μg/kg (Body weight), and triptolide dissolved in PBS was applied to mice through oral administration. Then, mice were scored starting from 10 weeks and then twice a week according to following standard: 0 (normal toe), 1 (acute inflammation including dactylitis), 2 (entheseal cell proliferation), 3 (cartilage formation), 4 (bone formation), and 5 (joint ankylosis). All toes were scored and calculated for each group. The experiments were approved by the Ethics Committee of our hospital.

### 2.21 Statistical analyses

All experiments in this study were conducted in triplicate, and the representative ones were exhibited. Statistical analyses were determined by Student's *t* test or one-way ANOVA, using SPSS version 19.0 software (IBM Corp., Armonk, NY, USA). Data were presented as the means ​± ​standard deviation (S.D.), and data considered significant at p-value < 0.05.

## 3 Results

### 3.1 Exosomes from ASMSCs had poorer effects on PBMCs osteoclast formation than exosomes from HDMSCs

**Supplementary Figure 1:**
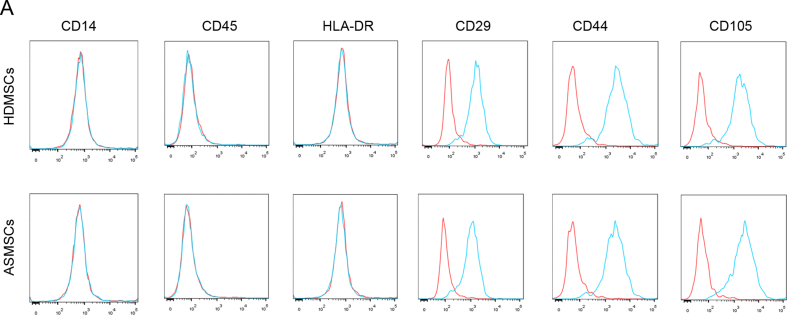
ASMSCs and HDMSCs were both negative for CD14, CD45 and HLA-DR and positive for CD29, CD44 and CD105. (A) The correlation between ASMSCs and HDMSCs on CD14, CD45, HLA-DR, CD29, CD44 and CD105.

**Supplementary Figure 2:**
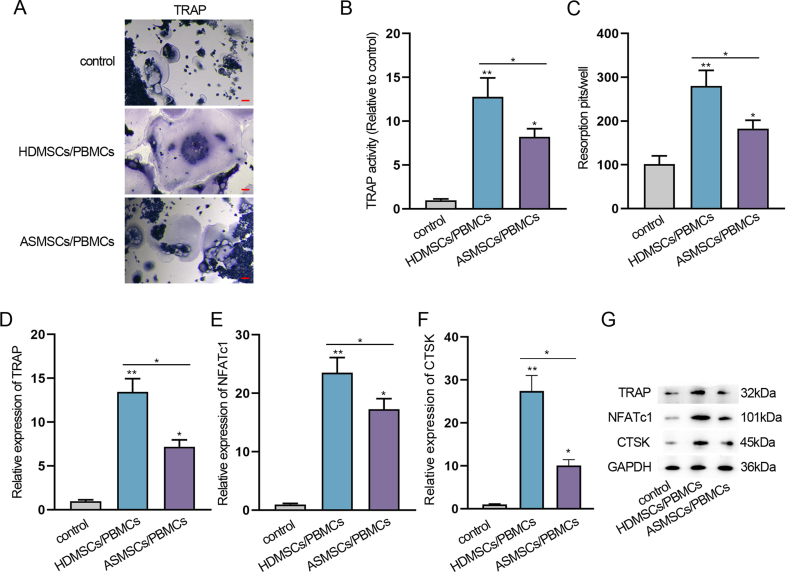
ASMSCs had poorer effects on PBMCs osteoclast formation than that of HDMSCs. (A) The cellular morphology in ASMSCs/PBMCs and HDMSCs/PBMCs was measured through TRAP staining. (B) TRAP activity was examined in ASMSCs/PBMCs and HDMSCs/PBMCs in comparison with the control group. (C) Numbers of resorption pits were counted in ASMSCs/PBMCs and HDMSCs/PBMCs. (D-G) The expression and protein levels of TRAP, NFATc1 and CTSK in ASMSCs/PBMCs and HDMSCs/PBMCs were measured by RT-qPCR as well as western blot. ∗P ​< ​0.05, ∗∗P ​< ​0.01.

ASMSCs and HDMSCs were collected from AS patients and healthy donors’ mesenchymal stem cells (MSCs). Both HDMSCs and ASMSCs possessed typical MSC surface markers. CD29, CD44 and CD105 presented positivity, while CD14, CD45 and HLA-DR were negative (Supplementary Fig. 1A). PBMCs were co-cultured with ASMSCs and HDMSCs, and then tartrate-resistant acid phosphatase (TRAP) was stained with co-cultured PBMCs with both ASMSCs and HDMSCs. It was shown that the staining intensity of HDMSCs was stronger than that of ASMSCs (Supplementary Fig. 2A). TRAP activity of co-cultured HDMSCs/PBMCs was strikingly higher than that of ASMSCs/PBMCs (Supplementary Fig. 2B). Meanwhile, the resorption pits were more in HDMSCs/PBMCs than in ASMSCs/PBMCs (Supplementary Fig. 2C). Moreover, the crucial transcription factor of osteoclast formation, nuclear factor of activated T-cells cytoplasmic (NFATc1), and the specific osteoclast markers TRAP and cathepsin K (CTSK), were all revealed to be augmented overtly in HDMSCs/PBMCs group in contrast to ASMSCs/PBMCs group (Supplementary Fig. 2D-G).

**Fig. 1:**
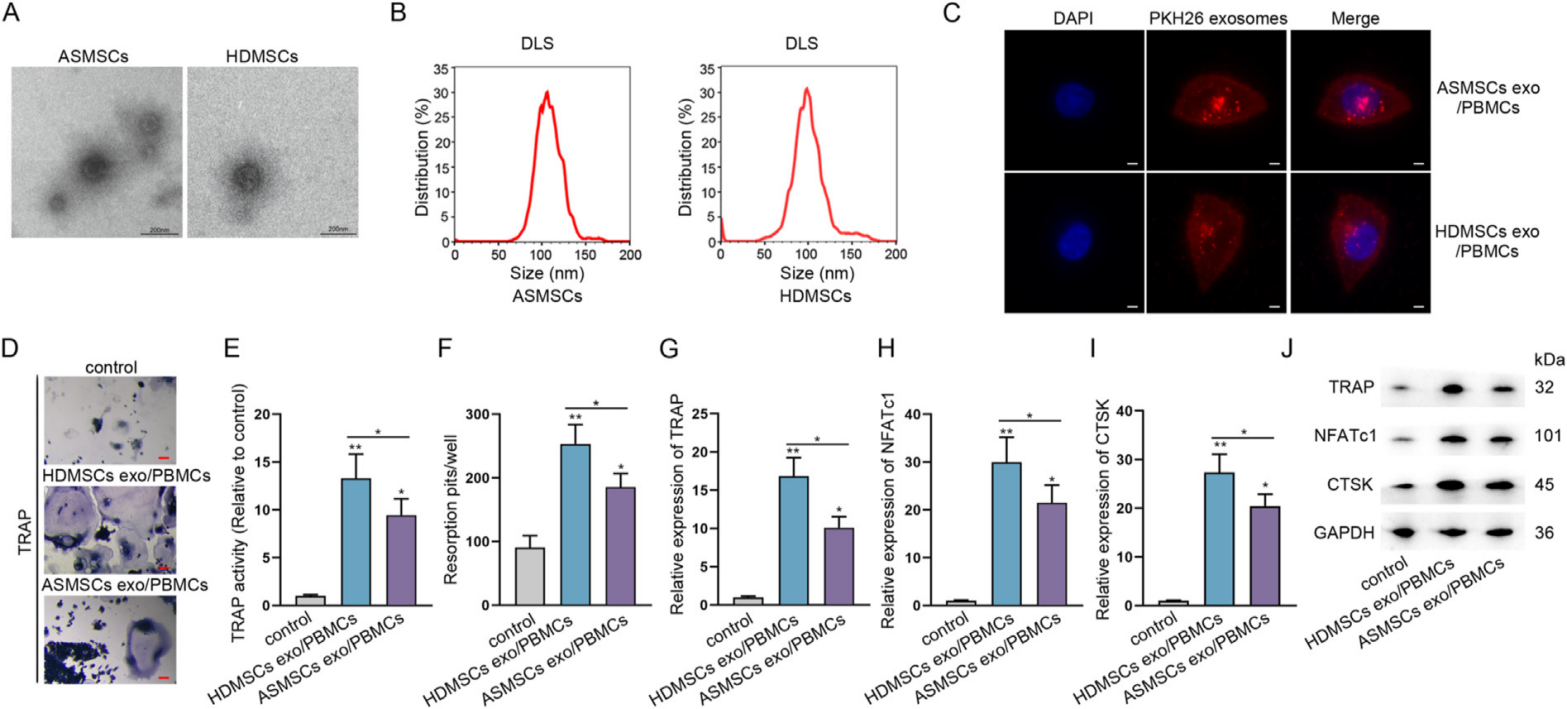
Exosomes from ASMSCs had poorer effects on PBMCs osteoclast formation than exosomes from HDMSCs (A) TEM images of exosomes derived from ASMSCs and HDMSCs (Bar ​= ​100 ​nm) (B) Exosomes absorbed by PBMCs were displayed by a confocal microscope (original magnification, ​× ​400). PBMCs were stained using DAPI. Scale bars, 10 ​μm (C) DLS analysis detected exosomes isolated from ASMSCs and HDMSCs (D) PBMCs were co-cultured with ASMSCs and HDMSCs-derived exosomes labeled PKH67 for 24 ​h (E) TRAP activity detection was examined in culture medium (F) Numbers of resorption pits were counted (G–J) RT-qPCR and western blot measured TRAP, NFATc1 and CTSK expression and protein levels. ∗P ​< ​0.05, ∗∗P ​< ​0.01.

Next, to ascertain whether ASMSCs and HDMSCs produced exosomes, we treated them with standard methods and separated exosomes. First, TEM validated the existence of exosomes from ASMSCs and HDMSCs (Fig. 1A) and NTA analysis showed the exosomes mainly had a size ranged from 50 to 150 ​nm (Fig. 1B). Further, it was discovered that PKH67-marked exosomes from either ASMSCs or HDMSCs could be successfully absorbed by PBMCs (Fig. 1C), and such cells were respectively renamed as HDMSCs exo/PBMCs and ASMSCs exo/PBMCs for further studies. It was shown that HDMSCs exo/PBMCs exhibited stronger TRAP staining, higher TRAP activity and more resorption pits than ASMSCs exo/PBMCs (Fig. 1D–F). At the same time, the expression and protein levels of TRAP, NFATc1 and CTSK were discovered to be higher in HDMSCs exo/PBMCs than in ASMSCs exo/PBMCs (Fig. 1G–J). To summarize, exosomes derived from HDMSCs had better effects on the osteoclast formation of PBMCs than exosomes derived from ASMSCs.

### 3.2 Exosomes from ASMSCs delivered more circ-0110634 to PBMCs than exosomes from HDMSCs

**Fig. 2:**
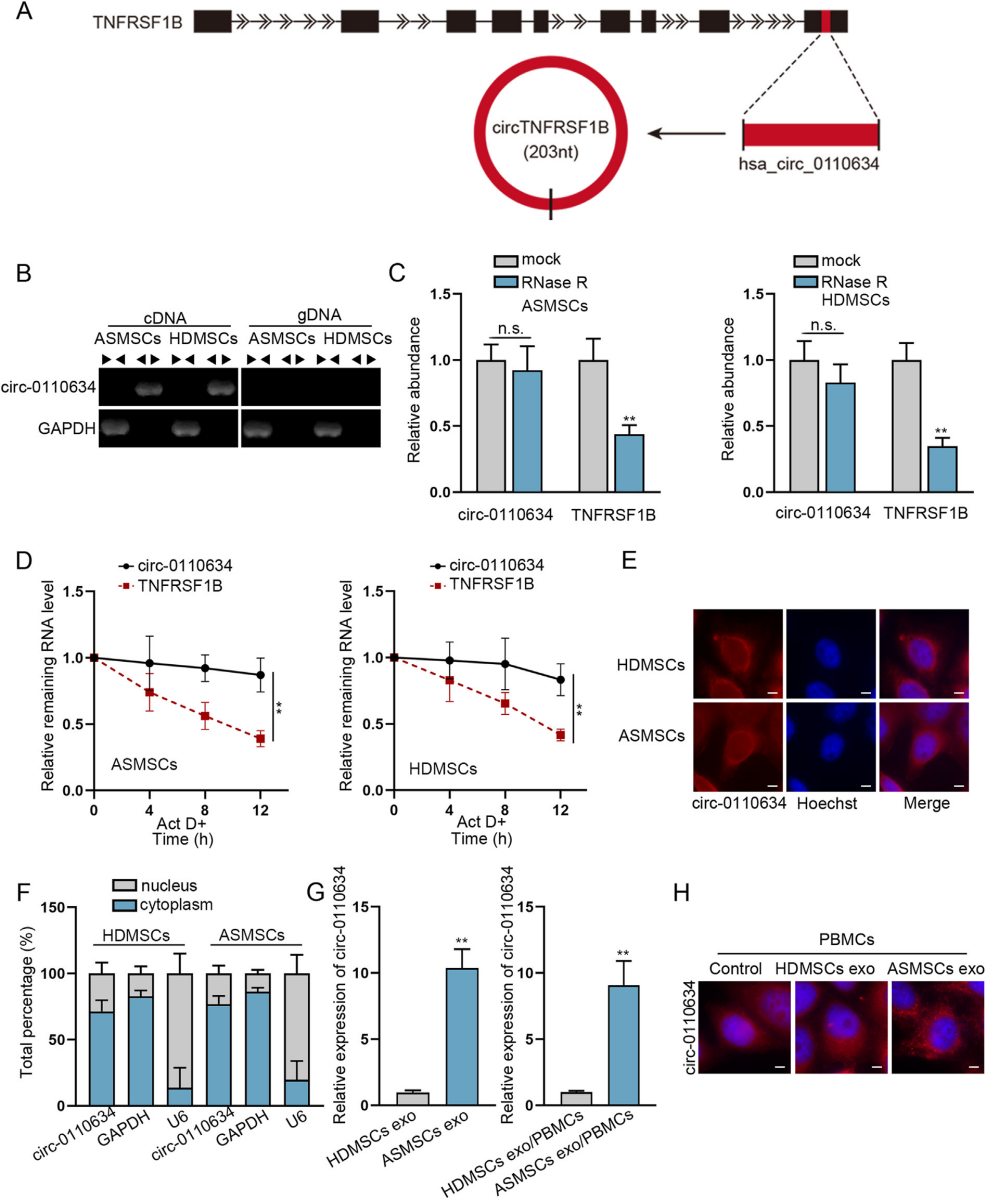
Circ-0110634 was up-regulated in ASMSCs exosomes/PBMCs cells (A) Schematic diagram of the genomic location and splicing pattern of circ-0110634 (B) Gel electrophoresis was performed to verify the circular structure of circ-0110634 (C–D) Rnase R and ActD treated with circ-0110634 and linear TNFRSF1B (E–F) FISH and Nuclear cytoplasm fractionation were carried out to verify circ-0110634 location in ASMSCs and HDMSCs (G) RT-qPCR was used to detect circ-0110634 expression in HDMSCs exosomes and ASMSCs exosomes and PBMCs co-cultured with HDMSC exosomes and ASMSCs exosomes (H) Circ-0110634 location in HDMSCs exosomes and ASMSCs exosomes was identified through FISH assay. ∗∗P ​< ​0.01, n. s. meant no significance.

**Supplementary Figure 3:**
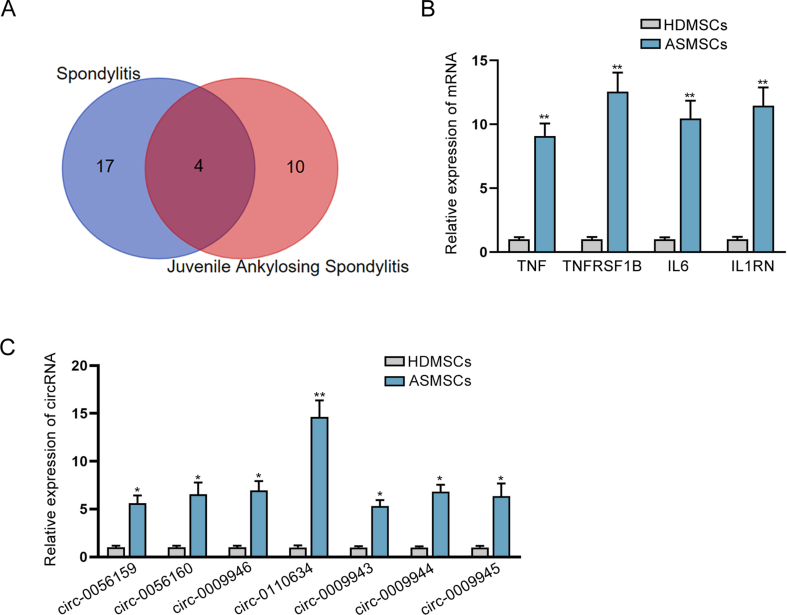
Circ-0110634 was overexpressed in ASMSCs. (A) Venn diagram showed the shared genes of spondylitis and juvenile AS. (B) RT-qPCR was utilized to examine mRNAs expression of the selected genes. (C) RT-qPCR was utilized to detect expression of circRNAs in ASMSCs and HDMSCs. ∗P ​< ​0.05, ∗∗P ​< ​0.01.

According to Malacards (https://www.malacards.org/) database, there were 4 shared genes of both spondylitis and juvenile AS, which were TNF, TNFRSF1B, IL6 and IL1RN (Supplementary Fig. 3A). RT-qPCR data further manifested that these four genes were significantly up-regulated in ASMSCs compared with HDMSCs (Supplementary Fig. 3B). Based on the data of circBase (http://www.circbase.org), only TNFRSF1B and IL1RN could generate circRNAs, and it was revealed that circ-0110634 was most evidently up-regulated in ASMSCs compared to HDMSCs (Supplementary Fig. 3C). Circ-0110634 is originated from the exon 9 of TNFRSF1B gene, with a length of 203bp (Fig. 2A). It manifested that circ-0110634 was only amplified by divergent primers in cDNA (Fig. 2B). Furthermore, we observed that circ-0110634 had no obvious changes while linear TNFRSF1B expression was decreased strikingly upon RNase R digestion (Fig. 2C). Simultaneously, circ-0110634 degraded much slower than linear TNFRSF1B when inhibiting RNA synthesis via ActD (Fig. 2D). By performing FISH and subcellular fractionation assays, circ-0110634 was determined to amass in the cytoplasm of ASMSCs and HDMSCs (Fig. 2E and F). Additionally, circ-0110634 expression was higher in ASMSCs exosomes than in HDMSCs exosomes, resulting in higher circ-0110634 level in ASMSCs exo/PBMCs than HDMSCs exo/PBMCs (Fig. 2G). Further, the results of FISH assays indicated more cytoplasmic accumulation of circ-0110634 in PBMCs treated with exosomes from ASMSCs (Fig. 2H). In a word, circ-0110634 was highly expressed in ASMSCs exo/PBMCs compared to HDMSCs exo/PBMCs.

### 3.3 Circ-0110634 inhibited the osteoclastogenesis of PBMCs

**Fig. 3:**
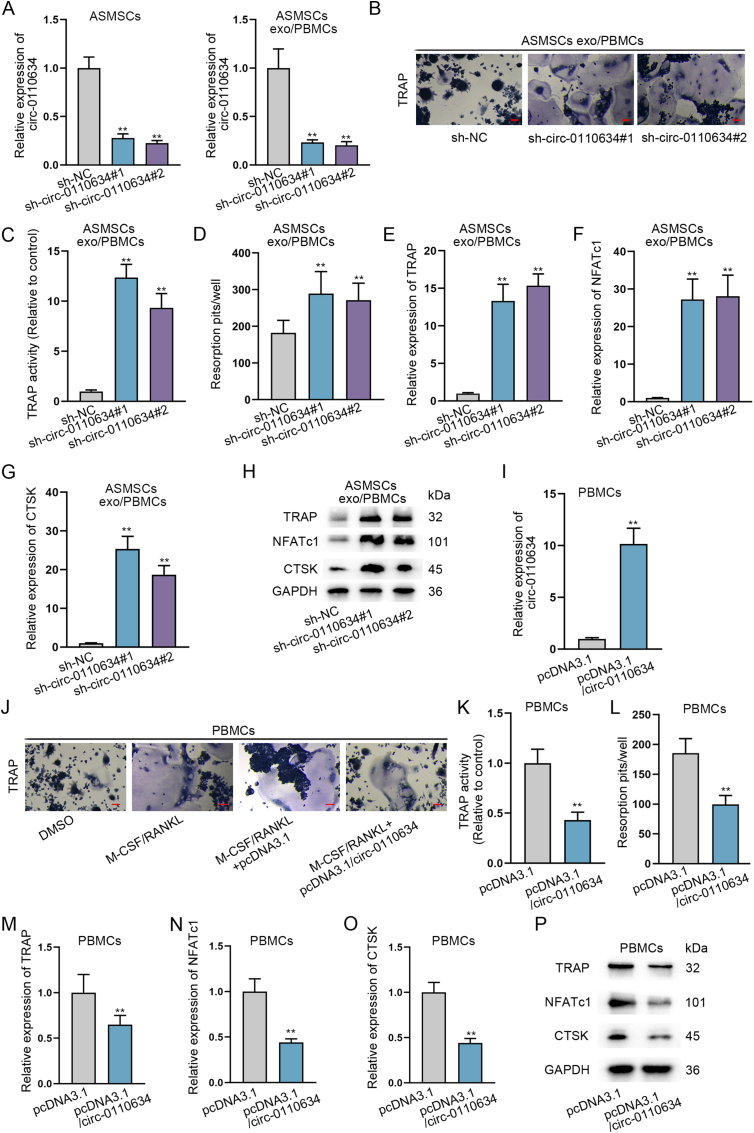
Circ-0110634 inhibited the osteoclastogenesis in ASMSCs exosomes/PBMCs (A) Circ-0110634 expression was knockdown by transfecting sh-circ-0110634#1/2 in ASMSCs and low expression of circ-0110634 was co-cultured with exo/PBMCs (B) The cellular morphology in ASMSCs exo/PBMCs was measured by TRAP staining (C) TRAP activity was examined in culture medium (D) Numbers of resorption pits were counted (E–H) The expression and protein levels of TRAP, NFATc1 and CTSK in ASMSCs treated with exo/PBMCs transfected with sh-circ-0110634 were analyzed by RT-qPCR and western blot (I) Circ-0110634 expression in PBMCs transfected with pcDNA3.1/circ-0110634 was examined via RT-qPCR (J) The cellular morphology in PBMCs transfected with pcDNA3.1 or pcDNA3.1/circ-0110634 was detected by TRAP staining after M-CSF/RANKL treatment (K) TRAP activity was examined in M-CSF/RANKL-treated PBMCs with different transfections (L) Numbers of resorption pits were counted in indicated PBMCs (M−P) RT-qPCR along with western blot was taken to analyze the expression and protein levels of TRAP, NFATc1 and CTSK in indicated PBMCs. ∗∗P ​< ​0.01.

Then, we continued to analyzed the effect of circ-0110634 on osteoclast formation of PBMCs. Hence, we applied sh-circ-0110634#1/2 to interfere circ-0110634 in ASMSCs, so as to lower circ-0110634 level in ASMSCs exo/PBMCs (Fig. 3A). It was then observed that down-regulation of circ-0110634 led to increased TRAP staining number (Fig. 3B), along with enhanced TRAP activity (Fig. 3C), augmented resorption pits (Fig. 3D), and increased expression of osteoclast markers, in ASMSCs exo/PBMCs (Fig. 3E–H). In the meantime, gain-of-function experiments were also carried out after validating the enhanced expression of circ-0110634 induced by pcDNA3.1/circ-0110634 transfection in PBMCs (Fig. 3I). PBMCs were co-treated with M-CSF and RANKL to induce osteoclast formation, and data revealed that circ-0110634 overexpression cut down the TRAP staining positivity and activity (Fig. 3J and K), as well as the bone resorption capacity (Fig. 3L). Moreover, the levels of TRAP, NFATc1 and CTSK were found to be lessened in face of circ-0010634 up-regulation in PBMCs (Fig. 3M−P). In short, circ-0110634 had inhibitory impacts on osteoclastogenesis.

### 3.4 Circ-0110634 bound to TNFRII and TRAF2

**Fig. 4:**
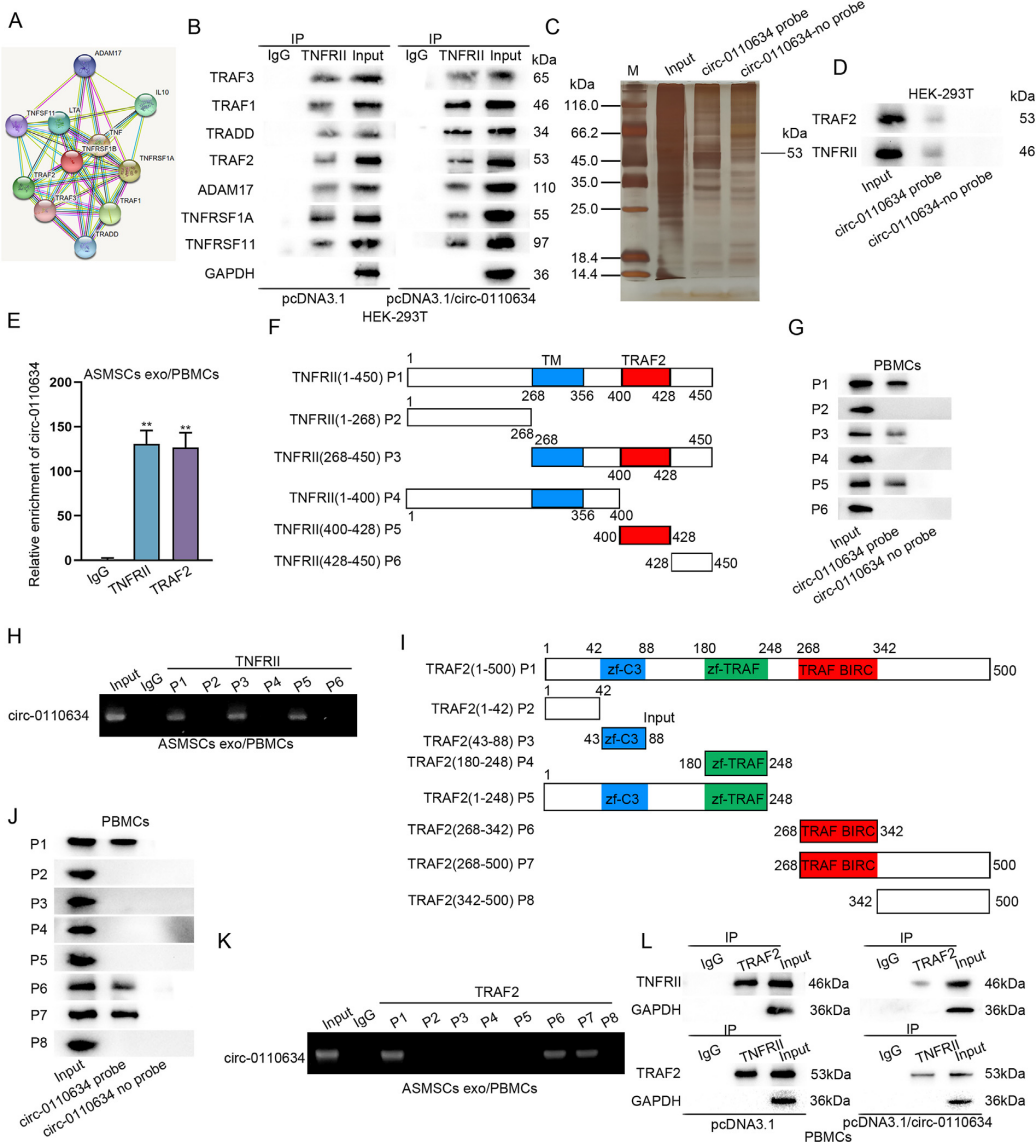
Circ-0110634 bound to TNFRII and TRAF2 (A) Potential proteins which could interact with TNFRII (B) The interaction between these proteins and TNFRII in HEK-293T cells with pcDNA3.1/circ-0110634 (C) RNA pull down assay was taken to verify whether circ-0110634 could combine with TRAF2 (D–E) RNA-protein pull-down and RIP assays were taken to verify whether circ-0110634 could bind to TRAF2 and TNFRII (F) TNFRII protein was cut down into 6 pieces (G–H) RNA-protein pull-down assays and gel electrophoresis were taken to verify the specific part circ-0110634 could bind to TNFRII (I) TRAF2 protein was cut into 8 pieces (J–K) RNA-protein pull down assays and gel electrophoresis were performed to confirm the binding capacity between specific pieces of TRAF2 and circ-0110634 (L) The binding capacity between TRAF2 and TNFRII was analyzed in circ-0110634-upregulated cells. ∗∗P ​< ​0.01.

**Supplementary Figure 4:**
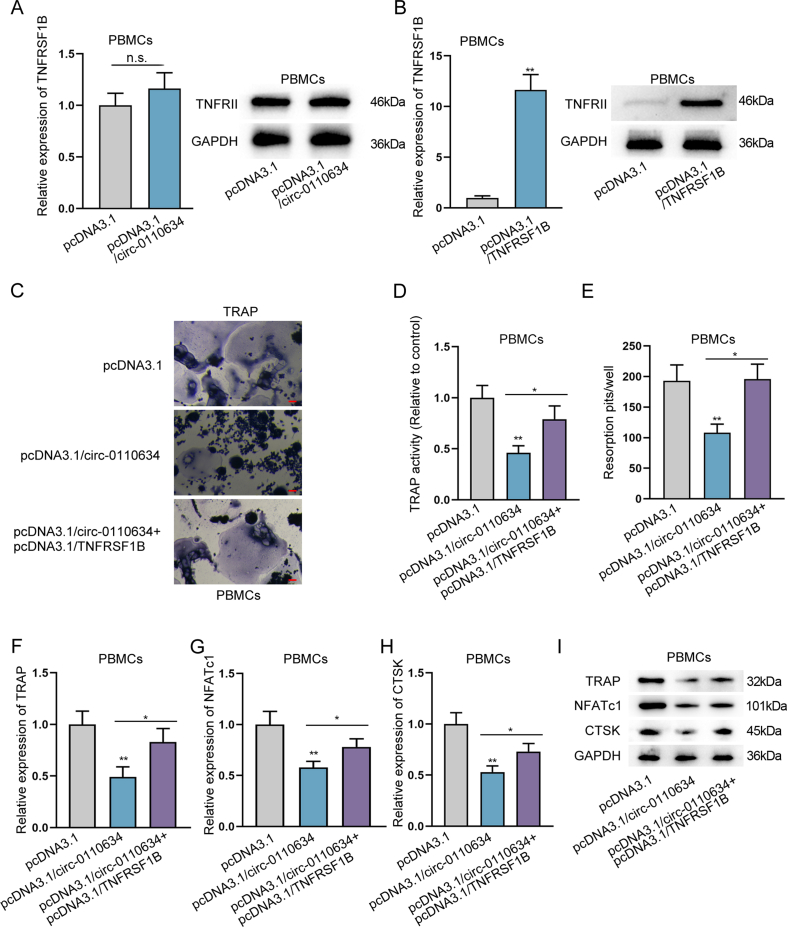
The inhibitory effect on the osteoclastogenesis caused by circ-0110634 overexpression was rescued by TNFRSF1B up-regulation. (A) The effects of circ-0110634 on TNFRSF1B and its protein were evaluated by RT-qPCR and western blot. (B) TNFRSF1B expression was enhanced by pcDNA3.1/TNFRSF1B in PBMCs. (C) The cellular morphology in PBMCs transfected with pcDNA3.1 or pcDNA3.1/circ-0110634 or pcDNA3.1/circ-0110634+pcDNA3.1/TNFRSF1B was measured through TRAP staining. (D) TRAP activity was examined in PBMCs transfected with pcDNA3.1 or pcDNA3.1/circ-0110634 or pcDNA3.1/circ-0110634+pcDNA3.1/TNFRSF1B. (E) Numbers of resorption pits were counted in PBMCs transfected with pcDNA3.1 or pcDNA3.1/circ-0110634 or pcDNA3.1/circ-0110634+pcDNA3.1/TNFRSF1B. (F–I) RT-qPCR along with western blot was taken to analyze the expression and protein levels of TRAP, NFATc1 and CTSK in PBMCs transfected with pcDNA3.1 or pcDNA3.1/circ-0110634 or pcDNA3.1/circ-0110634+pcDNA3.1/TNFRSF1B. ∗P ​< ​0.05, ∗∗P ​< ​0.01.

Subsequently, we tried to verify whether circ-0110634 may regulate its host gene TNFRSF1B to suppress the osteoclastogenesis. RT-qPCR and western blot data showed that up-regulation of circ-0110634 had no obvious impact on TNFRSF1B expression (Supplementary Fig. 4A). However, the restraining effects of circ-0110634 upregulation on PBMC osteoclastogenesis were offset by enhanced expression of TNFRSF1B (Supplementary Fig. 4B-I). Excluding the influence on TNFRSF1B/TNFRII expression, we guessed that circ-0110634 might regulate the function of TNFRII to affect osteoclastogenesis in PBMCs. In this regard, we found some proteins interacting with TNFRII vis STRING tool (https://cn.string-db.org/) (Fig. 4A). Interestingly, it was shown that up-regulating circ-0110634 mainly strengthened the interaction between TNFRII and TRAF2 in HEK-293T cells (Fig. 4B). Results of RNA pull down assay and mass spectrometry analysis further verified that TRAF2 could bind to circ-0110634 (Fig. 4C). Moreover, both data from RNA protein pull down and RIP assays validated that TRAF2 and TNFRII could bind to circ-0110634 (Fig. 4D and E). For next-step study, we divided TNFRII structure into 6 pieces and marked them as P1-6 (Fig. 4F). It was disclosed that circ-0110634 significantly bound to TNFRII at P1 (1–450), P3 (268–450) and P5 (400–428) (Fig. 4G and H). Similarly, we divided TRAF2 structure into 8 pieces, named P1–P8 (Fig. 4I), and observed that P1 (1–500), P6 (268–342) and P7 (268–500) of TRAF2 could bind to circ-0110634 (Fig. 4J and K). More importantly, Co-IP assay data validated that the interactivity between TRAF2 and TNFRII was attenuated upon circ-0110634 up-regulation (Fig. 4L). In conclusion, circ-0110634 bound to both TRAF2 and TNFRII to block TRAF2-TNFRII interaction in PBMCs.

### 3.5 Circ-0110634 accelerated TRAF2 self-ubiquitination and degradation

**Fig. 5:**
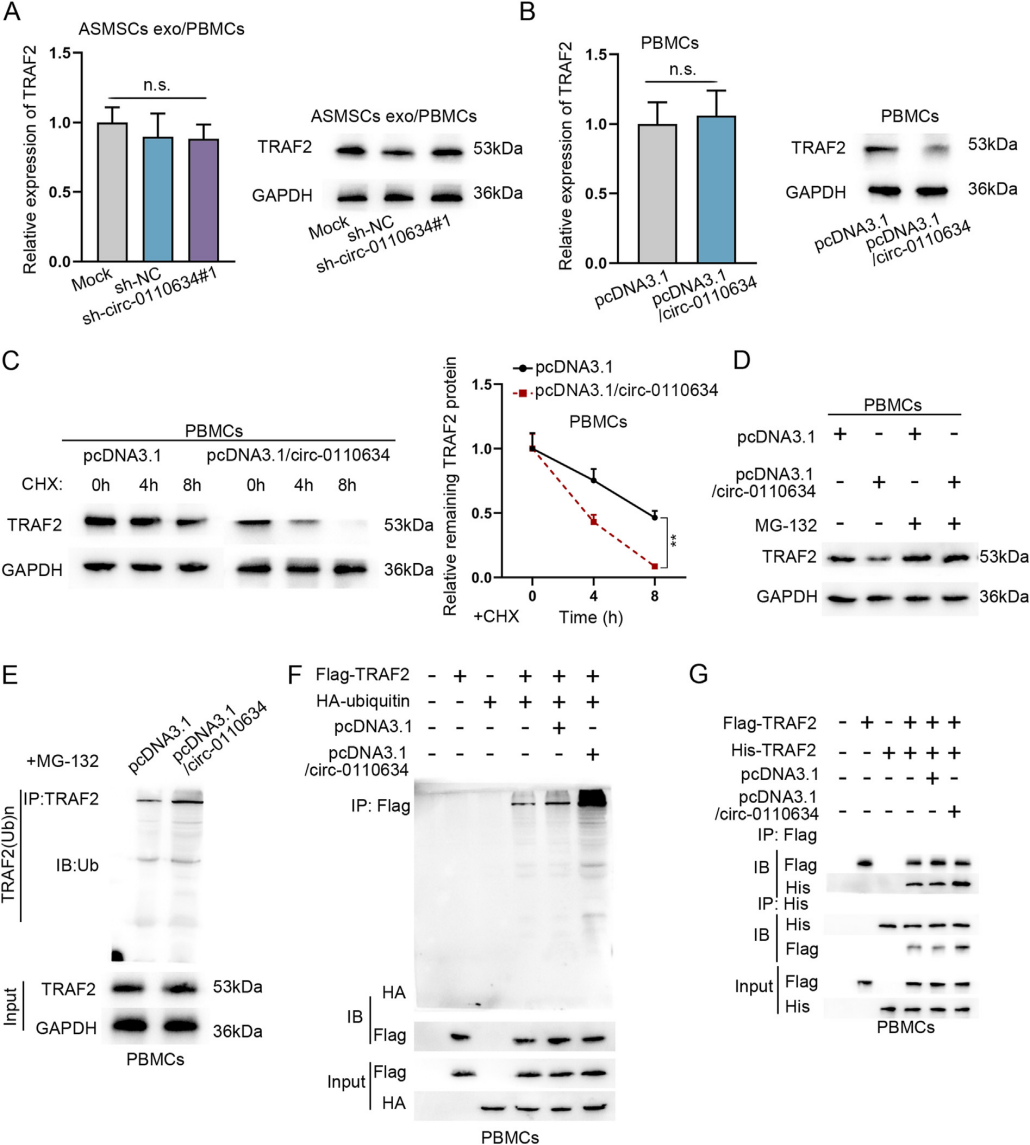
Circ-0110634 accelerated TRAF2 self-ubiquitination and degradation (A–B) RT-qPCR along with western blot was taken to examine TRAF2 expression and protein in cells with sh-circ-0110634#1 and pcDNA3.1/circ-0110634 (C–D) Western blot was taken to measure TRAF2 protein level by adding CHX and MG132 (E–F) Ubiquitination assays were carried out to measure TRAF2 ubiquitination under the conditions of PBMCs (G) Co-IP assay was taken to detect the interaction between dissociative TRAF2 and ubiquitin upon circ-0110634 up-regulation. ∗∗P ​< ​0.01, n. s. meant no significance.

Next, we analyzed the impact of circ-0110634 on TNFRII-bound TRAF2. It was showed that exosomes from ASMSCs evidently lowered the protein level of TRAF2, which was then reversed by circ-0110634 knockdown. Nevertheless, the mRNA expression of TRAF2 kept unchanged all the time (Fig. 5A). On the contrary, the protein level of TRAF2 was decreased by up-regulated circ-0110634 expression, without no apparent changes in TRAF2 mRNA (Fig. 5B). Then, we tested the regulation mechanism of circ-0110634 on TRAF2 protein. As shown in Fig. 5C, the half-life of TRAF2 under CHX treatment was shortened after circ-0110634 overexpression. Moreover, the treatment of MG-132 countervailed the inhibitory effect of circ-0110634 up-regulation on TRAF2 protein in PBMCs (Fig. 5D). As expected, we observed that the ubiquitination level of TRAF2 was elevated in response to circ-0110634 up-regulation (Fig. 5E). A further investigation indicated that the interaction between TRAF2 and ubiquitin was strengthened after circ-0110634 overexpression (Fig. 5F). We constructed His-TRAF2 and Flag-TRAF2 and then found that when circ-0110634 was up-regulated, the interaction between His-TRAF2 and Flag-TRAF2 was strengthened (Fig. 5G). To sum up, circ-0110634 promoted the ubiquitination and degradation of TRAF2.

### 3.6 Circ-0110634 repressed osteoclastogenesis via regulating TRAF2

**Fig. 6:**
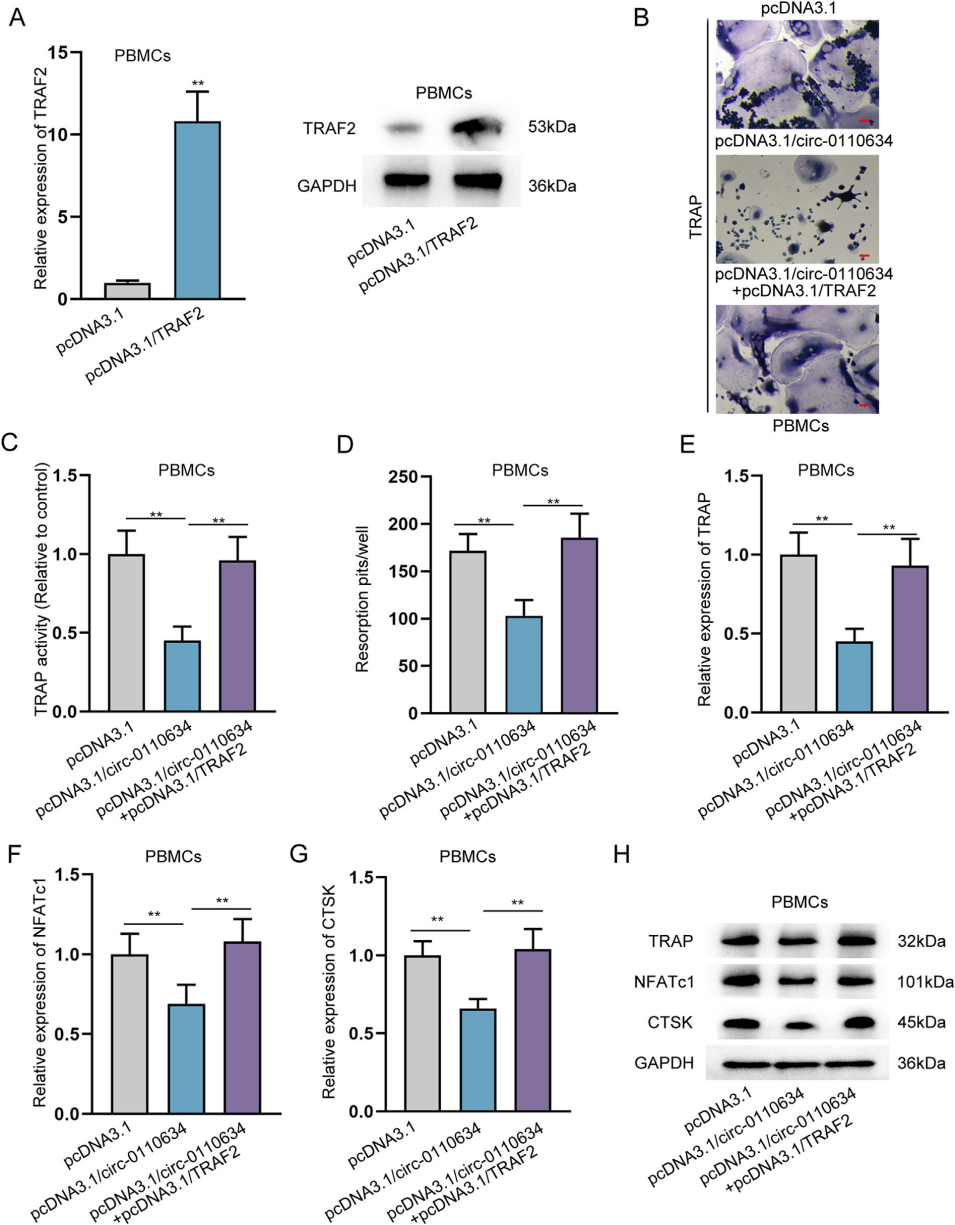
Circ-0110634 inhibited osteoclastogenesis via regulating TRAF2 (A) TRAF2 expression and protein was measured in cells transfected with pcDNA3.1/TRAF2 (B) The cellular morphology in PBMCs transfected with pcDNA3.1 or pcDNA3.1/circ-0110634 or pcDNA3.1/circ-0110634+pcDNA3.1/TRAF2 was measured through TRAP staining (C) TRAP activity was examined in PBMCs transfected with pcDNA3.1 or pcDNA3.1/circ-0110634 or pcDNA3.1/circ-0110634+pcDNA3.1/TRAF2 (D) Numbers of resorption pits were counted in PBMCs transfected with pcDNA3.1 or pcDNA3.1/circ-0110634 or pcDNA3.1/circ-0110634+pcDNA3.1/TRAF2 (E–H) RT-qPCR along with western blot was taken to analyze the expression and protein levels of TRAP, NFATc1 and CTSK in PBMCs transfected with pcDNA3.1 or pcDNA3.1/circ-0110634 or pcDNA3.1/circ-0110634+pcDNA3.1/TRAF2. ∗∗P ​< ​0.01.

**Supplementary Figure 5:**
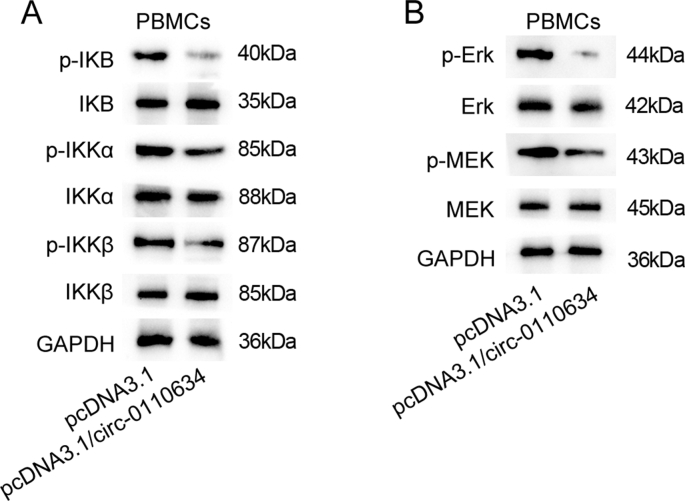
Circ-0110634 overexpression inactivated NF-κB and MAPK pathways. (A-B) Key proteins of NF-κB and MAPK pathways were measured in cells with up-regulation of circ-0110634.

Next, we aimed to validate whether circ-0110634 regulates TRAF2 to affect osteoclastogenesis in PBMCs. In this regard, we up-regulated TRAF2 expression by the transfection of pcDNA3.1/TRAF2 (Fig. 6A). It was discovered that the falling tendency of TRAP staining positivity and activity caused by circ-0110634 upregulation were reversed in response to of TRAF2 overexpression (Fig. 6B and C), so was that of resorption pits (Fig. 6D). Likewise, the repressed expression levels of TRAP, NFATc1 and CTSK resulted from overexpression of circ-0110634 were counteracted responding to the up-regulation of TRAF2 (Fig. 6E–H). Thereafter, we examined the influence ofcirc-0110634 on two well-known TRAF2 downstream pathways, NF-κB and MAPK pathways in PBMCs. It was unveiled that the levels of p-IKB, p-IKKα, p-IKKβ, p-Erk and p-MEK were lessened after the overexpression of circ-0110634 (Supplementary Fig. 5A and B). To conclude, circ-0110634 inhibited osteoclastogenesis by regulating TRAF2 to inactivate the NF-κB and MAPK pathways.

### 3.7 Triptolide promoted osteoclastogenesis via inhibiting circ-0110634 expression

**Fig. 7:**
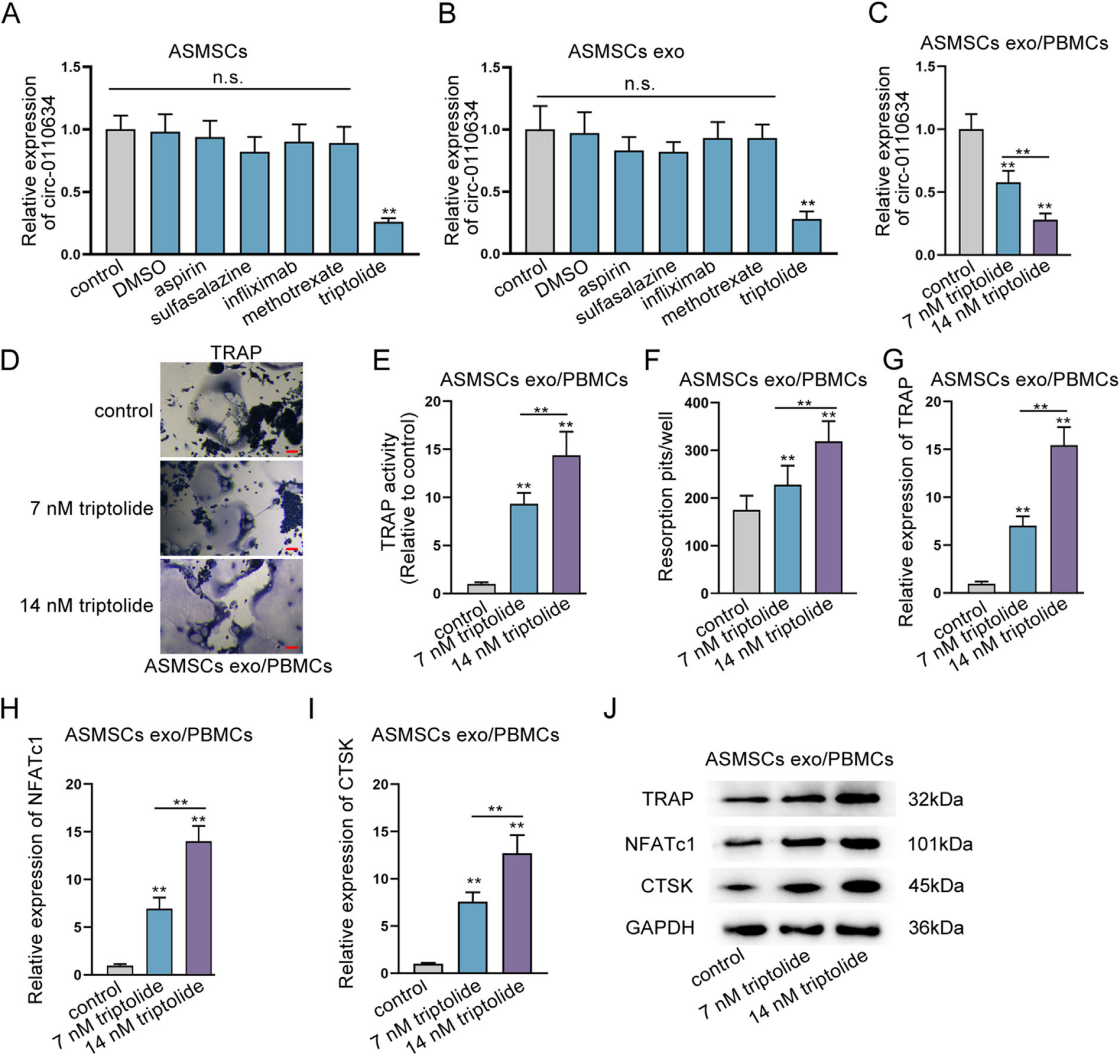
Triptolide promoted osteoclastogenesis via inhibiting circ-0110634 (A–B) RT-qPCR was taken to examine several medicines effects on circ-0110634 expression in ASMSCs and its exosomes (C) Circ-0110634 expression was examined in different doses of triptolide applied in cells (D) The cellular morphology in ASMSCs exo/PBMCs treated with control or 7 ​nm triptolide or 14 ​nm triptolide was measured through TRAP staining (E) TRAP activity was examined in ASMSCs exo/PBMCs treated with control or 7 ​nm triptolide or 14 ​nm triptolide (F) Numbers of resorption pits were counted in ASMSCs exo/PBMCs treated with control or 7 ​nm triptolide or 14 ​nm triptolide (G–J) RT-qPCR along with western blot was taken to analyze the expression and protein levels of TRAP, NFATc1 and CTSK in ASMSCs exo/PBMCs treated with increasing doses of triptolide. ∗∗P ​< ​0.01, n. s. meant no significance.

**Supplementary Figure 6:**
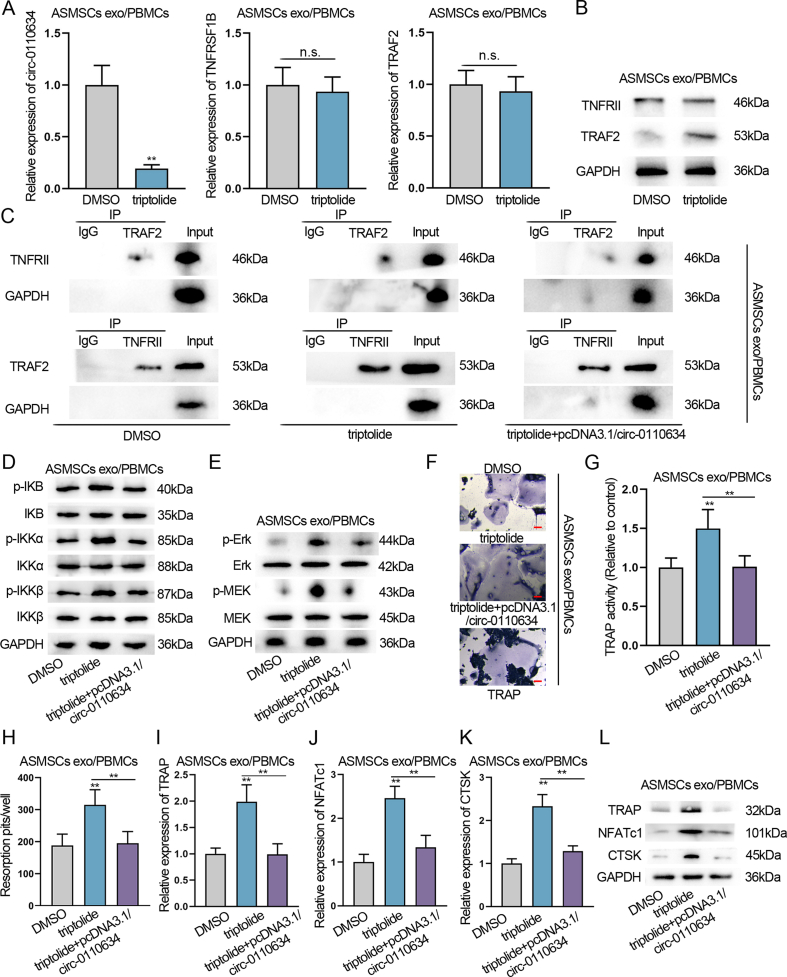
Triptolide promoted osteoclastogenesis by inhibiting circ-0110634 to inactivate the NF-κB and MAPK pathways. (A-B) Effects of triptolide on circ-0110634, TNDRSF1B, TRAF2 and TNFRII were examined by RT-qPCR and western blot. (C) Co-IP assays were carried out to detect the combination between TRAF2 and TNFRII affected by triptolide or triptolide ​+ ​pcDNA3.1/circ-0110634. (D-E) Western blot was taken to examine the expression of key factors in the NF-κB and MAPK pathways by triptolide or triptolide ​+ ​pcDNA3.1/circ-0110634. (F) The cellular morphology in ASMSCs exo/PBMCs transfected with triptolide or triptolide ​+ ​pcDNA3.1/circ-0110634 was measured through TRAP staining. (G) TRAP activity was examined in ASMSCs exo/PBMCs transfected with triptolide or triptolide ​+ ​pcDNA3.1/circ-0110634. (H) Numbers of resorption pits were counted in ASMSCs exo/PBMCs transfected with triptolide or triptolide ​+ ​pcDNA3.1/circ-0110634. (I-L) RT-qPCR along with western blot was taken to analyze the expression and protein levels of TRAP, NFATc1 and CTSK in ASMSCs exo-treated PBMCs under different conditions. ∗∗P ​< ​0.01, n.s. meant no significance.

**Supplementary Figure 7:**
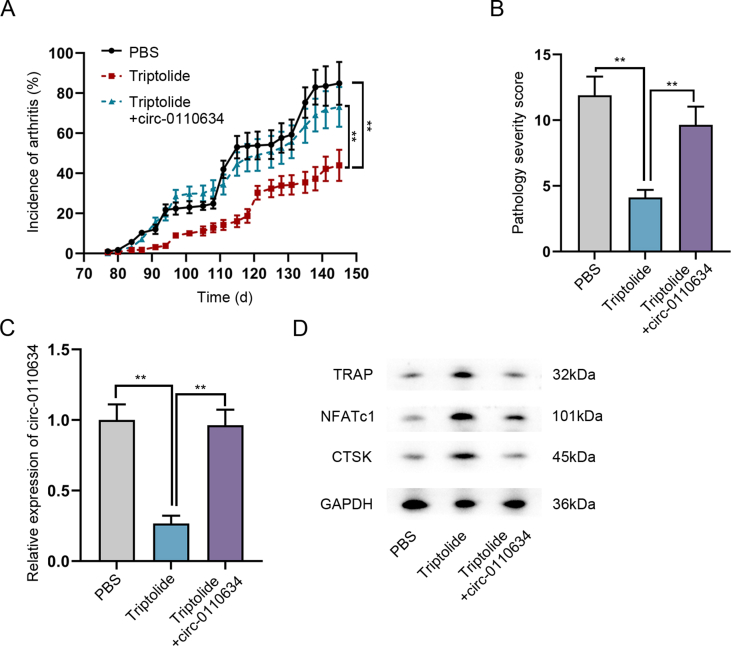
Triptolide attenuated arthritis in DBA/1 mice model through targeting circ-0110634. (A-B) Cumulative incidence of arthritis and pathology severity score in DBA/1 mice with different treatments. (C) Circ-0110634 expression in injured joints from indicated groups of DBA/1 mice was measured by RT-qPCR. (D) The protein expression of TRAP, NFATc1 and CTSK in indicated mice joint tissues was measured by western blot analysis. ∗∗P ​< ​0.01.

Several medicines which are usually used to treat AS are applied to treat ASMSCs. As indicated in Fig. 7A, only triptolide significantly decreased circ-0110634 expression in ASMSCs, while other medicines had no such distinct effect. The same result was also seen in exosomes derived from ASMSCs under indicated drug treatment (Fig. 7B). Further, we discovered that triptolide could cut down circ-0110634 expression in ASMSCs exo/PBMCs in a dose-dependent way (Fig. 7C). Accordingly, triptolide elicited dose-dependent promoting impacts on TRAP staining, TRAP activity, and bone resorption capacity (Fig. 7D–F). The same results were also observed in the expression of osteoclast markers including TRAP, NFATc1 and CTSK (Fig. 7G–J). In depth, we then intended to determine whether triptolide may promote osteoclastogenesis through circ-0110634/TNFRSF1B/TRAF2 axis. We found that triptolide decreased circ-0110634 expression but failed to affect the mRNA levels of TNFRSF1B and TRAF2 (Supplementary Fig. 6A), therefore enhancing TRAF2 protein level without no effect on TNFRII protein (Supplementary Fig. 6B). Data of Co-IP assays displayed that triptolide strengthened the interaction between TRAF2 and TNFRII, but this interaction was weakened when circ-0110634 was up-regulated (Supplementary Fig. 6C). Moreover, triptolide was found to activate NF-κB and MAPK pathways, but this capacity was inhibited upon circ-0110634 overexpression (Supplementary Fig. 6D and E). Furthermore, it manifested that circ-0110634 up-regulation restored the enhanced effects of triptolide on TRAP staining, TRAP activity, bone resorption as well as the levels of TRAP, NFATc1 and CTSK (Supplementary Fig. 6F-L). For further verification, spontaneous arthritis model was constructed via DBA/1 mice. It was shown that the addition of triptolide mitigated the severity of arthritis, and its effect was countervailed by the further transfection of pcDNA3.1/circ-0110634 (Supplementary Fig. 7A and B). In addition, above phenomena were shown to be attributed to circ-0110634 upregulation induced recoveries of circ-0110634 expression and osteoclastogenesis changed by triptolide treatment (Supplementary Fig. 7C and D). In short, triptolide facilitated osteoclastogenesis via targeting circ-0110634 expression.

### 3.8 Graphical abstract illustrating the regulatory mechanism of triptolide in the osteoclastogenesis of ASMSCs exosomes-treated PBMCs

**Fig. 8:**
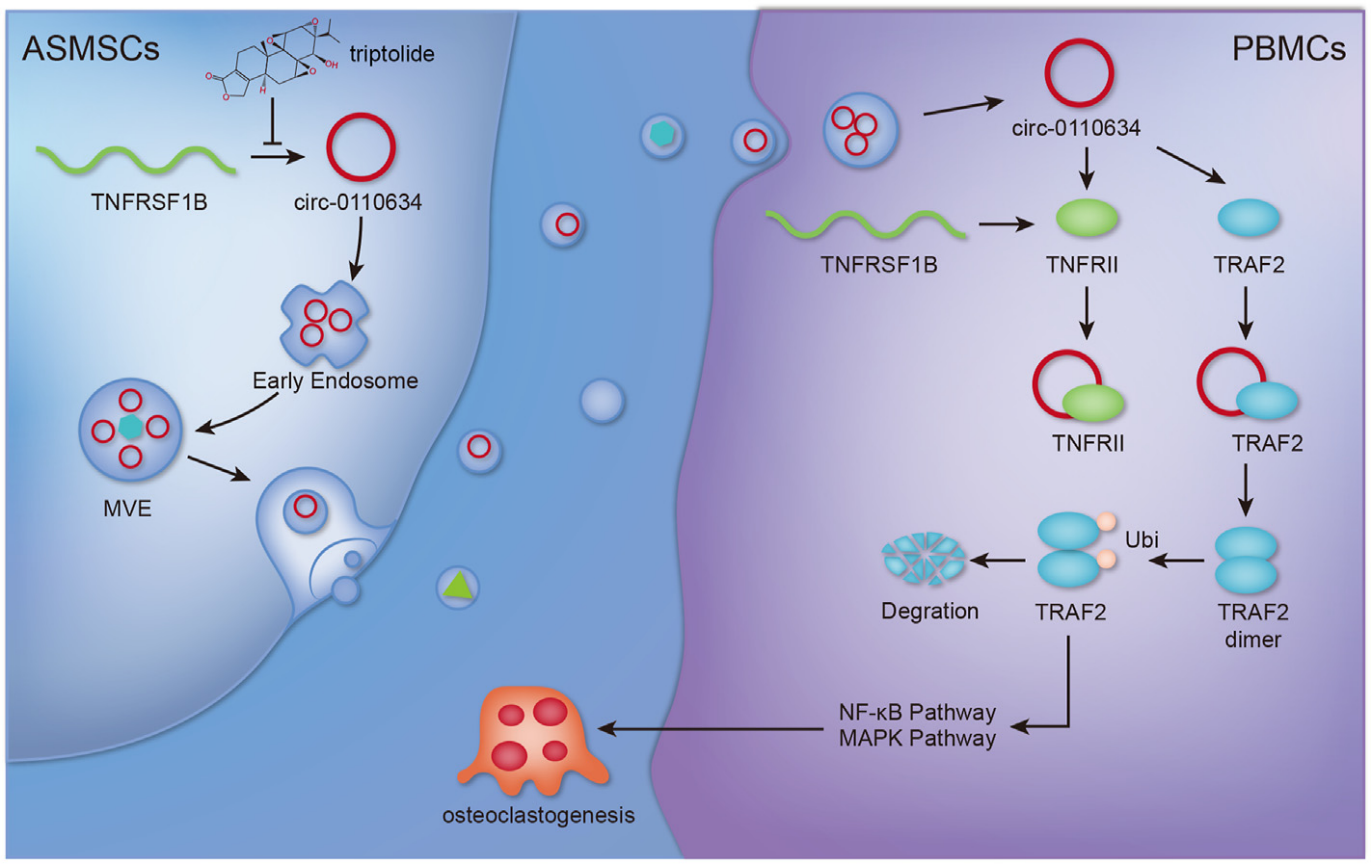
Graphical abstract illustrated the regulatory mechanism of how triptolide regulated exosomal circ-0110634 from ASMSCs in the osteoclastogenesis of PBMCs.

In our study, we elucidated that circ-0110634 suppressed the osteoclastogenesis by interacting with TRAF2 and TNFRII to accelerate TRAF2 dimerization, ubiquitination and degradation, thereby inactivating NF-κB and MAPK pathways. Then, triptolide promoted the osteoclastogenesis of PBMCs via decreasing the exosomal transference of circ-0110634 from ASMSCs (Fig. 8).

## 4 Discussion

AS is a sort of rheumatic disease which causes inflammatory back pain and structural and functional changes in axial skeleton leading to bad quality of life [24]. Osteoclast has crucial functions in bone resorbing and formation [25]. Based on previous references, although the number of osteoclasts in AS patients is equivalent to that in healthy donors, osteoclast differentiating genes were low-expressed in AS patients [26]. Presently, our study found that PBMCs co-cultured with ASMSCs exhibited reduced capacity of osteoclastogenesis compared to those co-cultured with HDMSCs.

Exosomes are derived from various types of bone cells such as mesenchymal stem cells, osteoblasts, and osteoclasts, which play substantial roles in bone remodeling processes including osteoclastogenesis [[27], [28], [29]]. Former research has also illustrated that exosomes from MSCs take part in the modulation of osteoclastogenesis *in vitro* [30]. In this work, we uncovered that a circRNA derived from TNFRSF1B, circ-0110634, was significantly up-regulated in ASMSCs and exosomes from ASMSCs compared to HDMSCs and HDMSCs exosomes. Moreover, we testified that circ-0110634 played a inhibitory part in osteoclastogenesis and ASMSCs delivered exosomal circ-0110634 to restrained the osteoclastogenesis of PBMCs. Similar to the role of hsa_circ_0002715 and hsa_circ_0035197 in patients with new-onset rheumatoid arthritis [31], our findings also provide a base for circ-0110634 as a promising target for patients with AS.

TNFRII and TRAF2 are necrosis factor receptors belonging to TNF family [32]. Interestingly, we found that circ-0110634 could combine with both TNF-RII and TRAF2. In addition, TNF-RII and c-IAP1 mediate ubiquitination and degradation of TRAF2 [33]. Besides, TRAF6 dimerization has been shown to be essential for the assembly of K63-linked ubiquitin chains and important for its auto-ubiquitination [34,35]. TRAF2 is a ubiquitin ligase like TRAF6, and it can also form a dimer [36]. Here, we found that circ-0110634 accelerated the dimerization of TRAF2 to induce TRAF2 auto-ubiquitination and degradation, so as to reduce TRAF2 level in PBMCs. For all we know, TRAF2 along with PI3 kinase (PI3K) induces phosphorylation of Akt, resulting in persistent nuclear factor-κB (NF-κB) activation [37], as well as subsequent activation of MAPK [38]. Unsurprisingly, it was disclosed through our study that circ-0110634 could inactivate the NF-κB and MAPK pathways through suppressing TRAF2. More importantly, the suppressive effect of circ-0110634 on the osteoclastogenesis of PBMCs could be reversed by TRAF2 overexpression. All these results suggested that circ-0110634 impaired osteoclastogenesis through inactivating TRAF2-regulated NF-κB and MAPK pathways.

Triptolide is a compound isolated from a Chinese medicinal herb, which possesses potent antitumor, immunosuppressive, and anti-inflammatory properties in treating human diseases [39]. Current literature have revealed the inhibitory effect of triptolide on osteoclastogenesis mainly in bone lytic diseases [40,41]. Nevertheless, it has also been reported that triptolide inhibits osteogenesis in AS which is characterized by excessive bone formation [42], while there are bidirectional interactions between osteoblasts and osteoclasts in bone formation [43]. Opposite to the inhibition of triptolide on osteogenesis discovered by Ji, W., et al. [42], our data supported the dose-dependent promotion of triptolide on osteoclastogenesis. Further, our study found that triptolide could activate the NF-κB and MAPK pathways to facilitate the osteoclastogenesis through decreasing circ-0110634. Another study has discovered that triptolide represses the function of TNF-α in osteoblast differentiation through NF-κB signaling pathway [44]. More significantly, spontaneous arthritis model established in DBA/1 mice proved that triptolide treatment mitigated the severity of arthritis by suppressing circ-0110634. All these data supported that triptolide might alleviate osteoclastogenesis in AS via targeting circ-0110634.

In conclusion, our study suggested that triptolide attenuates osteoclastogenesis in AS through inhibiting exosomal transfer of circ-0010634 from ASMSCs to PBMCs. However, our study also contains a few drawbacks. For example, we did not examine the downstream factors of both NF-κB pathway and MAPK pathway, which requires further in-depth researches in the near future. Besides, more clinical analyses are needed for further evidencing the findings in present work.

## Funding

This study was supported by Science and technology project of Jiangsu Provincial Bureau of Traditional Chinese Medicine under grant (No.ZD201804) and Peak Talent Project of Jiangsu Hospital of Traditional Chinese Medicine under grant (No.y2018rc10).

## Ethic approval

The human and animal experiments were approved by the Ethical Committee of Affiliated Hospital of Nanjing University of Chinese Medicine.

## Declaration of competing interest

The authors have no conflicts of interest relevant to this article.
